# The prognosis prediction significance of Hounsfield unit value for stroke patients treated by intravenous thrombolysis

**DOI:** 10.1186/s12880-021-00592-6

**Published:** 2021-04-07

**Authors:** Zhengqi Zhu, Ru Zhang, Kaixuan Ren, Ruochen Cong, Xiangyang Zhu, Li Zhu, Tianle Wang

**Affiliations:** 1 Department of Radiology, The Second Affiliated Hospital of Nantong University, 6 Haierxiang North Road, Nantong, 226001 Jiangsu China; 2 Department of Neurology, The Second Affiliated Hospital of Nantong University, Nantong, 226001 China

**Keywords:** Hounsfield unit value, Intravenous thrombolysis, Stroke, Prognosis

## Abstract

**Background:**

Intravenous thrombolysis (IVT) is a rapid and effective treatment in the early stage of ischemic stroke patients and the purpose of this work is to explore the significance of Hounsfield unit (HU) value in Alberta Stroke Program Early CT Score (ASPECTS) for predicting the clinical prognosis of stroke patients with middle cerebral artery occlusion (MCAO) treated by IVT.

**Methods:**

The 84 stroke patients with MCAO treated by IVT were divided into good prognosis group (48 cases) and poor prognosis group (36 cases). HU ratio and HU difference calculated from non-contrast computed tomography between groups were analyzed.

**Results:**

The HU ratio of good prognosis group was higher than that in poor prognosis group and the HU difference of good prognosis group was lower than that in poor prognosis group (*P* < 0.05). The HU ratio and ASPECTS were negatively correlated with the infarct volume, and the HU difference was positively correlated with the infarct volume (*P* < 0.05). HU difference was an independent risk factor for prognosis of patients with MCAO treated by IVT. The area under the receiver operating characteristic curve of HU ratio and HU difference for prognosis was 0.743 and 0.833 respectively.

**Conclusion:**

The HU value changes are related to the clinical prognosis of stroke patients with MCAO treated by IVT, HU value may be a prognostic indicator for stroke patients with MCAO treated by IVT.

## Background

Intravenous thrombolysis (IVT) is a rapid and effective treatment in the early stage of ischemic stroke patients, which can restore blood flow, improve cerebral blood circulation, and improve the clinical prognosis of patients. However, the efficacy of IVT in different patients is significant different, and their clinical outcomes are different. Previous studies have shown that patients with higher Alberta Stroke Program Early CT Score (ASPECTS) usually have better clinical outcomes and a lower risk of symptomatic intracranial hemorrhage [1–3].

ASPECTS is a scoring method that can be used to evaluate early ischemic changes in the middle cerebral artery blood supply area. ASPECTS can be obtained on CT scan. This method is simple and easy to use, and is widely used by many stroke centers. However, stroke patients treated with IVT are in the hyperacute stage of stroke, and their head CT images change very slight, and the consistency and accuracy of ASPECTS are questioned due to subjective differences of observers [1].

The HU value reflects the water uptake of the infarcted tissue. As the water uptake increases, the HU value decreases. HU value can reflect the length of infarction time and can be used to judge the stroke time of the patients [4, 5]. Previous studies have shown that HU value from non-contrast computed tomography (NCCT) in ASPECTS helps to identify the core of infarction and is related to the final infarct volume of stroke after IVT [6, 7]. Therefore, this research aimed to explore the significance of HU value in ASPECTS for predicting the clinical prognosis of stroke patients with middle cerebral artery occlusion (MCAO) treated by IVT.

## Methods

### Patients

One hundred and forty stroke patients with MCAO in our hospital who received IVT from January 2018 to December 2019 were retrospectively analyzed. Inclusion criteria: ① age ≥ 18 years old; ② patients who arrived at the hospital within 6 h after stroke onset and received IVT; ③ stroke patient with MCAO was diagnosed by MRI images within 7 days after IVT; ④ complete patient clinical data. Exclusion criteria: ① poor CT image quality; ② patients with bilateral infarction. The last, 84 patients were included in the study and divided into good prognosis group (48 cases) and poor prognosis group (36 cases). This study was approved by the Research Ethics Committee of the Second Affiliated Hospital of Nantong University. The severity of stroke at admission was evaluated using National Institute of Health stroke scale (NIHSS). The clinical prognosis at 3 months after thrombolysis was evaluated using the modified Rankin Scale (mRS). According to mRS, the 84 stroke patients were divided into good prognosis group (0–2, 48 cases) and poor prognosis group (3–6, 36 cases). The clinical data of patients was showed in the Table 1.

**Table 1:** Patients clinical characteristics

	Good prognosis group (n = 48)	Poor prognosis group (n = 36)	*P*
Age, mean ± SD	70.6 ± 11.69	72.3 ± 8.46	0.458
*Gender*			0.051
Male	29 (60.4%)	22 (61.1%)	
Female	19 (39.6%)	14 (38.9%)	
*Infarct side*			0.705
Left	26 (54.2%)	18 (50%)	
Right	22 (45.8%)	18 (50%)	
Symptom onset to initial head CT, mean ± SD	120.3 ± 70.12	141.4 ± 84.85	0.216
*Initial NIHSS, median (range)*	7 (2–35)	14 (4–35)	< **0.001**
HBP	26(54.1%)	18(50%)	0.705
DM	15 (31.2%)	7 (19.4%)	0.223
AF	10 (20.8%)	11 (30.5%)	0.309
ASPECTS, median (range)	10 (8–10)	9 (5–10)	< **0.001**
*Hemorrhagic transformation*	9 (18.8%)	16 (44.4%)	**0.011**
HMCAS	9 (18.8%)	14 (38.9%)	**0.041**
LNO	3 (6.2%)	8 (22.2%)	**0.032**
Infarct volume, median (range)	3.61(0.12–82.10)	79.42(6.99–285.26)	< **0.001**
HU ratio, mean ± SD	9.97 ± 0.19	9.75 ± 0.27	< **0.001**
HU difference, mean ± SD	11.14 ± 2.44	17.81 ± 7.61	< **0.001**

Bold values indicate the *P* < 0.05

*AF* atrial fibrillation, *ASPECTS* Alberta Stroke Program Early CT Score, *DM* diabetes mellitus, *HBP* high blood pressure, *HMCAS* hyperdense middle cerebral artery sign, *HU* Hounsfield unit, *LNO* lenticular nucleus obscuration, *NIHSS* National Institute of Health stroke scale

### Image analysis

Head scan was performed with Siemens FORCE CT (SOMATOM Force, Siemens Health Care, Germany). Scanning position: supine position; scanning positioning reference line: orbito-meatal line (OML); scanning parameter setting: tube voltage was 100 kV, tube current was 120 mA, and layer thickness was 2 mm. All patients underwent whole-brain MRI with the Siemens Verio 3.0 T MRI system (Magnetom Verio, Siemens Health Care, Germany). The scan settings for DWI using the SE echo-planar imaging (SE-EPI) sequence were as follows: TR = 6600 ms, TE = 100 ms, FA = 90°, FOV = 230 mm × 100 mm, matrix = 192 × 192, slice thickness = 5 mm, interslice gap = 1 mm, and b = 0, 1000 s/mm^2^.

**Fig. 1:**
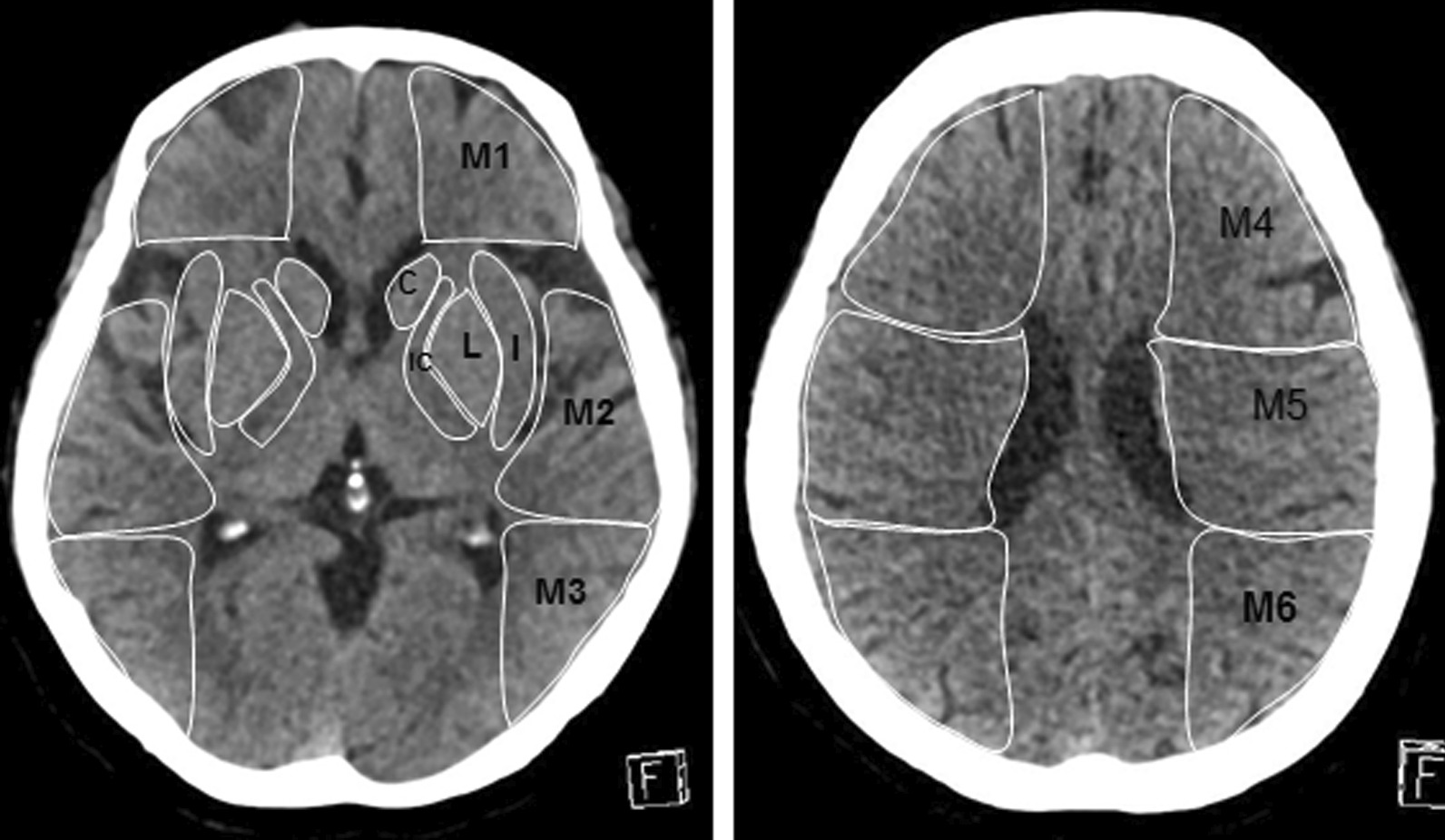
Ten ASPECTS regions were outlined on the NCCT images. HU ratio in ten ASPPECTS regions, C: 41.75/41.81, M1: 39.99/38.31, M4: 39.41/39.82, L: 41.82/41.29, M2: 42.11/44.54, M5: 37.26/37.72, IC: 31.67/34.03, M3: 41.19/38.85, M6: 38.95/39.04, I: 35.17/40.82. HU difference in ten ASPPECTS regions, C: |41.75–41.81|, M1: |39.99–38.31|, M4: |39.41–39.82|, L: |41.82–41.29|, M2: |42.11–44.54|, M5: |37.26–37.72|, IC: |31.67–34.03|, M3: |41.19–38.85|, M6: |38.95–39.04|, I: |35.17–40.82|

Two radiologists with more than 5 years of neuroimaging diagnosis experience who were blinded to the clinical data measured the NCCT images. Ten separate regions corresponding to the standard middle cerebral artery territory ASPECTS areas were manually outlined. Both investigators defined ROIs limits individually and a consensus read was done to obtain definite ROIs limits that were used for further analysis (Fig. 1). The imaging software (Syngo.via VB20 workstation) calculated the radiological density of each ASPECT region using Hounsfield Units (HU). At least three measurements were carried out in each region and the mean HU values for each ASPECT region were recorded.

The HU value of 10 ASPECTS area of the infarct side cerebral hemisphere was divided by the HU value of the corresponding ASPECTS area of the healthy side cerebral hemisphere to obtain 10 HU ratios and the HU ratio of each patient was obtained by adding the ratio of each area. The HU value of 10 ASPECTS area in the infarcted side cerebral hemisphere was subtracted from the HU value of the corresponding ASPECTS area of the healthy hemisphere to obtain 10 HU differences and the HU difference of each patient was obtained by adding the difference of each area.

The HU values were built up as follows: (a) HU density of one ROI of an ASPECT region (Average of pixel values across ROI) ≥ ROI Mean value plus error, e.g. SD. (b) At least three measurements in each regions, i.e. 3 or more ROI values, and mean HU values for each region ≥ 3 * REGION Mean plus error * (ROI Mean + SD). (c) Adding the 10 HU difference values or ratio values of each area per patient to generate patient value. ≥ Sum((3*REGION 1 Mean plus error * (ROI Mean + SD)), (3*REGION 2 Mean plus error * (ROI Mean + SD)), …, (3*REGION 10 Mean plus error * (ROI Mean + SD))) = HU Difference or ratio per patient + error. The HU difference or ratio per patient without error was used subsequent research and analysis according to previous studies [7–9].

Patient with hemorrhagic transformation was defined as hemorrhage found on MRI images within 7 days after thrombolytic therapy. The patient's cerebral infarct volume was calculated on the DWI image within 7 days after thrombolytic therapy. The perimeter of the area of abnormal high-signal intensity was traced on each DWI image. The total lesion volume was calculated as the sum of the infarct area on each DWI slice × (slice thickness + interslice gap).The unit of infarct volume is ml [10].

Baseline NCCT image was used to grade the ASPECTS standardized 10-point scale by two stroke neurologist. Two raters achieved one common ASPECTS score per patient through consensus during a joint reading session.

### Statistical analysis

The measurement data was expressed by mean ± SD or median, and the count data was expressed by percentage. The KS test was used to determine whether the data was normally distributed. The normal distribution data was analyzed with t test. Mann–Whitney U test was used to analyze non-normally distributed data. Spearman correlation analysis was used to evaluate the relationship between HU ratio/HU difference and infarct volume. The factors such as age, gender, initial NIHSS, ASPECTS, hyperdense middle cerebral artery sign (HMCAS), lenticular nucleus obscuration (LNO), HU ratio and HU difference were taken into the Binomial Logistic regression equation. The area under the ROC curve (AUC) was used to evaluate the diagnostic efficacy of ASPECTS, HU ratio and HU difference for the prognosis of stroke patients with MCAO treated by IVT. *P* value < 0.05 was considered statistically significant. All data were performed by SPSS 25.0 (IBM. Corp., Armonk, NY, USA) and MedCalc (MedCalc, Mariakerke, Belgium).

## Results

### Comparison of clinical features between the two groups

The good prognosis group had lower admission NIHSS score than the poor prognosis group (median 7 vs 14) (*P* < 0.05), and higher ASPECTS than the poor prognosis group (median 10 vs 9) (*P* < 0.05). The good prognosis group had a smaller infarct volume than the poor prognosis group (median 3.61 vs 79.42) (*P* < 0.05). The good prognosis group has a lower risk of hemorrhagic transformation than the poor prognosis group (18.8% vs 44.4%) (*P* < 0.05). We found that the HU ratio and HU difference between the two groups were significantly different. The good prognosis group had a higher HU ratio than the poor prognosis group (9.97 ± 0.19 vs 9.75 ± 0.27) (*P* < 0.05), and a lower HU difference than the poor prognosis group (11.14 ± 2.44 vs 17.81 ± 7.61) (*P* < 0.05). In addition, our study also found that early image signs of NCCT (HMCAS and LNO) were associated with the prognosis of thrombolytic patients. The detailed results were shown in Table 1.

### The relationship between HU ratio/HU difference/ ASPECTS and infarct volume

**Fig. 2:**
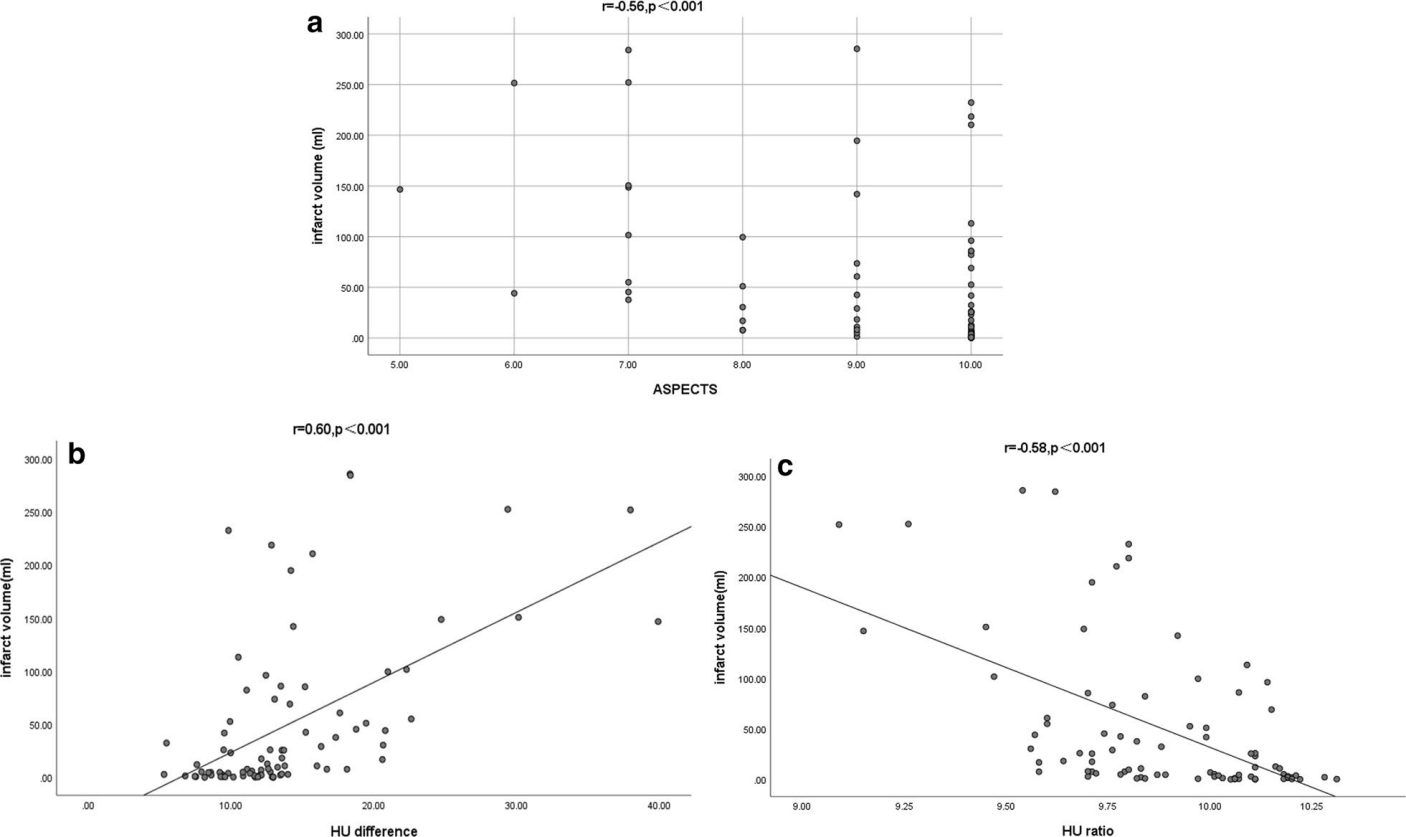
The relationship between HU ratio/HU difference/ASPECTS and infarct volume. **a** The ASPECTS was negatively correlated with the infarct volume, **b** the HU difference was positively correlated with the infarct volume, **c** the HU ratio was negatively correlated with the infarct volume

The HU ratio and ASPECTS were negatively correlated with the infarct volume, and the HU difference was positively correlated with the infarct volume (*P* < 0.05) (Fig. 2). The detailed results were shown in Table 2.

**Table 2:** The relationship between HU ratio/HU difference and infarct volume

	Spearman *r*	95% CI	*P*
HU ratio	− 0.58	(− 0.71, − 0.43)	< **0.001**
HU difference	0.60	(0.43, 0.72)	< **0.001**
ASPECTS	− 0.56	(− 0.67, − 0.39)	< **0.001**

Bold values indicate the *P* < 0.05

*ASPECTS* Alberta Stroke Program Early CT Score, *HU* Hounsfield unit

### Independent risk factors for prognosis of stroke patients with MCAO treated by IVT

The factors such as age, gender, initial NIHSS, ASPECTS, HMCAS, LNO, HU ratio and HU difference were taken into the Binomial Logistic regression equation. The results showed the initial NIHSS and HU difference was independent risk factors for prognosis of stroke patients with MCAO treated by IVT (*P* < 0.05). The detailed results were shown in Table 3.

**Table 3:** Independent risk factors for prognosis of stroke patients with MCAO treated by IVT

	B	Wald X^2^	OR	95% CI	*P*
Age	− 0.006	0.036	0.99	(0.93, 1.05)	0.850
Gender	0.455	0.453	1.57	(0.42, 5.93)	0.501
Initial NIHSS	0.116	6.335	1.12	(1.02, 1.22)	**0.012**
ASPECTS	− 0.682	0.880	0.51	(0.12, 2.10)	0.348
HMCAS	0.331	0.186	1.39	(0.31, 6.24)	0.666
LNO	− 1.465	1.047	0.23	(0.01, 3.82)	0.306
HU ratio	0.034	0.001	1.03	(0.03, 35.26)	0.985
HU difference	0.296	3.977	1.35	(1.00, 1.80)	**0.046**

Bold values indicate the *P* < 0.05

*ASPECTS* Alberta Stroke Program Early CT Score, *HMCAS* hyperdense middle cerebral artery sign, *HU* Hounsfield unit, *LNO* lenticular nucleus obscuration, *NIHSS* National Institute of Health stroke scale

### Diagnostic efficacy for the prognosis of stroke patients with MCAO treated by IVT

**Fig. 3:**
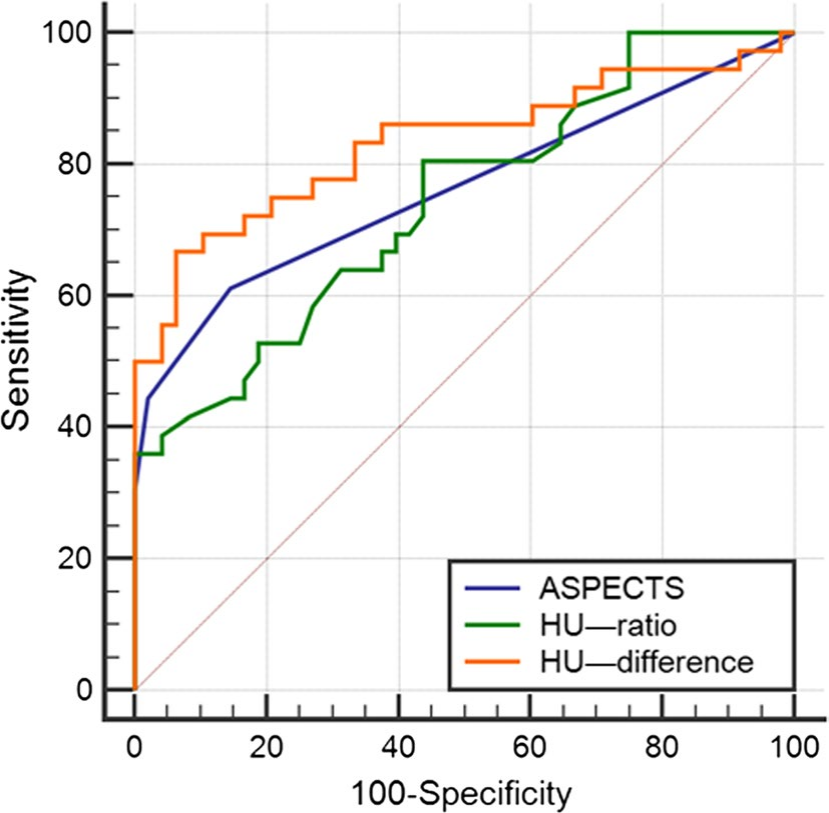
The AUC of ASPECTS, HU ratio and HU difference

The ROC curve showed that AUC of ASPECTS, HU ratio, HU difference were 0.762, 0.743 and 0.833, which were meaningful to predict the prognosis of patients (*P* < 0.05). The AUC of the HU difference was greater than that of ASPECTS and HU ratio, but no statistical significance was found (*P* > 0.05) (Fig. 3). The detailed results were shown in Table 4.

**Table 4:** The AUC of ASPECT S, HU ratio and HU difference for the prognosis of stroke patients treated by IVT

	AUC	*P*	Youden index	Cutoff value	Sensitivity %	Specificity %
ASPECTS	0.762	< **0.001**	0.465	9.00	61.11	85.42
HU ratio	0.743	< **0.001**	0.368	10.00	80.56	56.25
HU difference	0.833	< **0.001**	0.604	13.97	66.67	93.75

*ASPECTS* Alberta Stroke Program Early CT Score, *HU* Hounsfield unit

## Discussion

Our research found that the changes in HU value of ASPECTS were related to the clinical prognosis of stroke patients with MCAO treated by IVT. HU difference was the independent risk factor for prognosis of stroke patients with MCAO treated by IVT. The changes in HU value can be used to predict the clinical prognosis of stroke patients with MCAO treated by IVT. HU value measurement can help clinicians make quick decisions and select potential beneficiaries of IVT.

Our research found that the HU ratio of the good prognosis group was higher than that of the poor prognosis group, and the HU difference was lower than that of the poor prognosis group. This may be because the decrease in HU value is significantly related to the increase in water content of ischemic brain tissue. It was reported that increasing the water content of the cerebral hemisphere by 1% will cause the HU value to decrease by 1.8 HU. Water uptake content of infarcted brain tissue was related to the degree of infarction [11, 12]. Our research found the HU ratio was negatively correlated with the infarct volume, and the HU difference was positively correlated with the infarct volume. Smaller HU ratio and larger HU difference were related to larger infarct volume. HU value is also related to the state of cerebral blood flow. Kucinski et al. [13] reported that within 4.5 h after the onset of stroke, the decrease in HU value was related to the decrease in cerebral blood flow and volume. In the hyperacute phase of stroke, the HU value changes very slightly. Compared with CTP and MRI, NCCT is difficult to accurately delineate the infarct range and measure the HU value. However, for stroke centers without advanced imaging techniques such as CTP and MRI, NCCT is still the preferred imaging examination before thrombolytic therapy. In order to overcome this difficulty, we selected 10 ASPECT regions to measure HU values in stroke patients with MCAO. For the hyperacute phase of stroke, NCCT can detect brain tissue swelling and gray matter lesions in time. NCCT has the advantages of fast imaging speed, low price and wide popularity. NCCT does not require contrast injection and will not cause adverse reactions. Our study also found that early image signs of NCCT (hyperdense middle cerebral artery sign, lenticular nucleus obscuration) were associated with the prognosis of thrombolytic patients, which is consistent with previous studies [14–16].

**Fig. 4:**
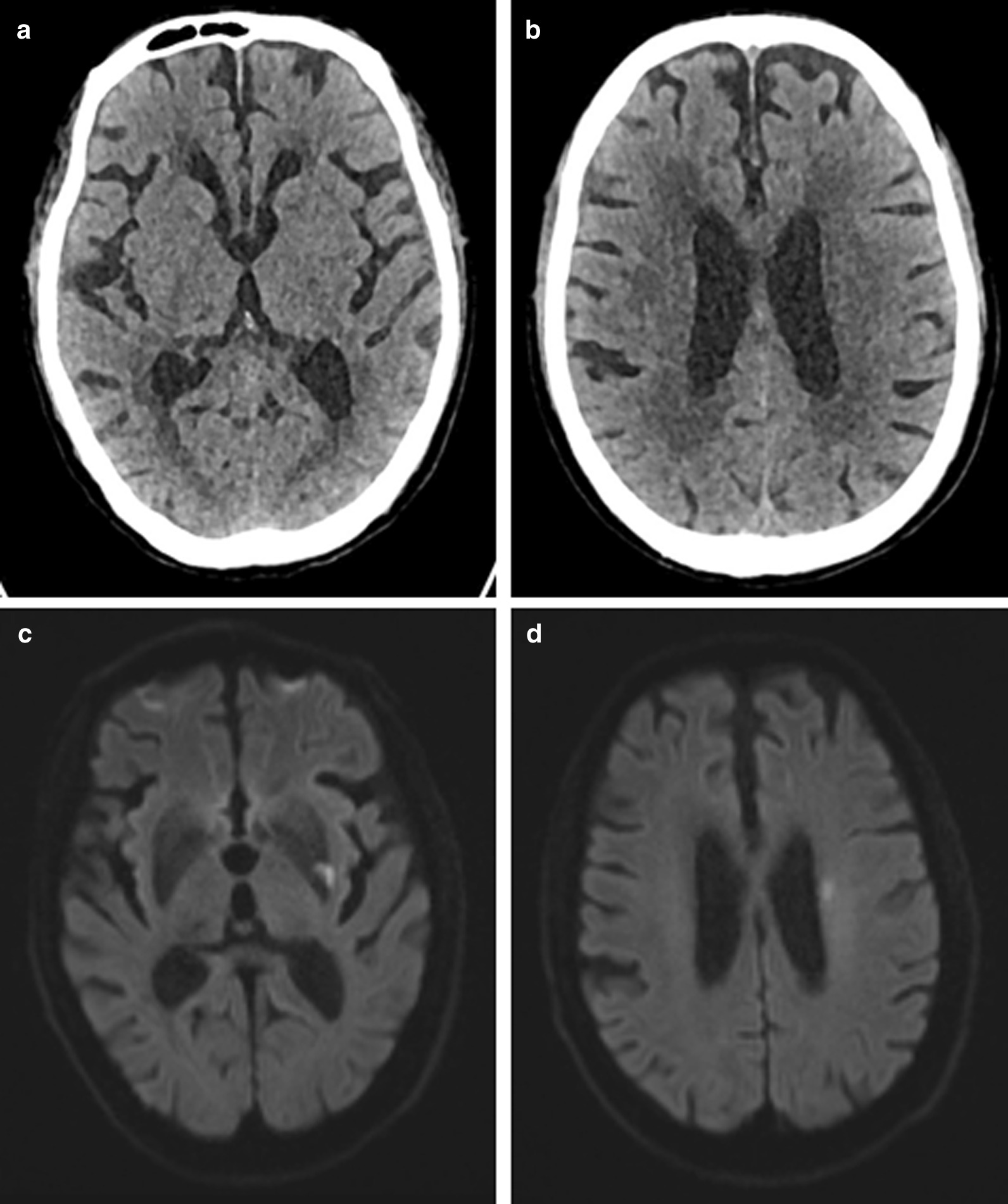
Patients with good clinical prognosis: a patient was admitted to the hospital 30 min after the onset of stroke. At admission, the patient's ASPECTS was 9 points, and the HU difference was 12.94. The infarct volume calculated on the DWI image at 7 days after thrombolysis was 4.14, and the 3-month mRS score was 2 points. **a**, **b** NCCT image on admission. **c**, **d** DWI image at 7 days after thrombolysis

**Fig. 5:**
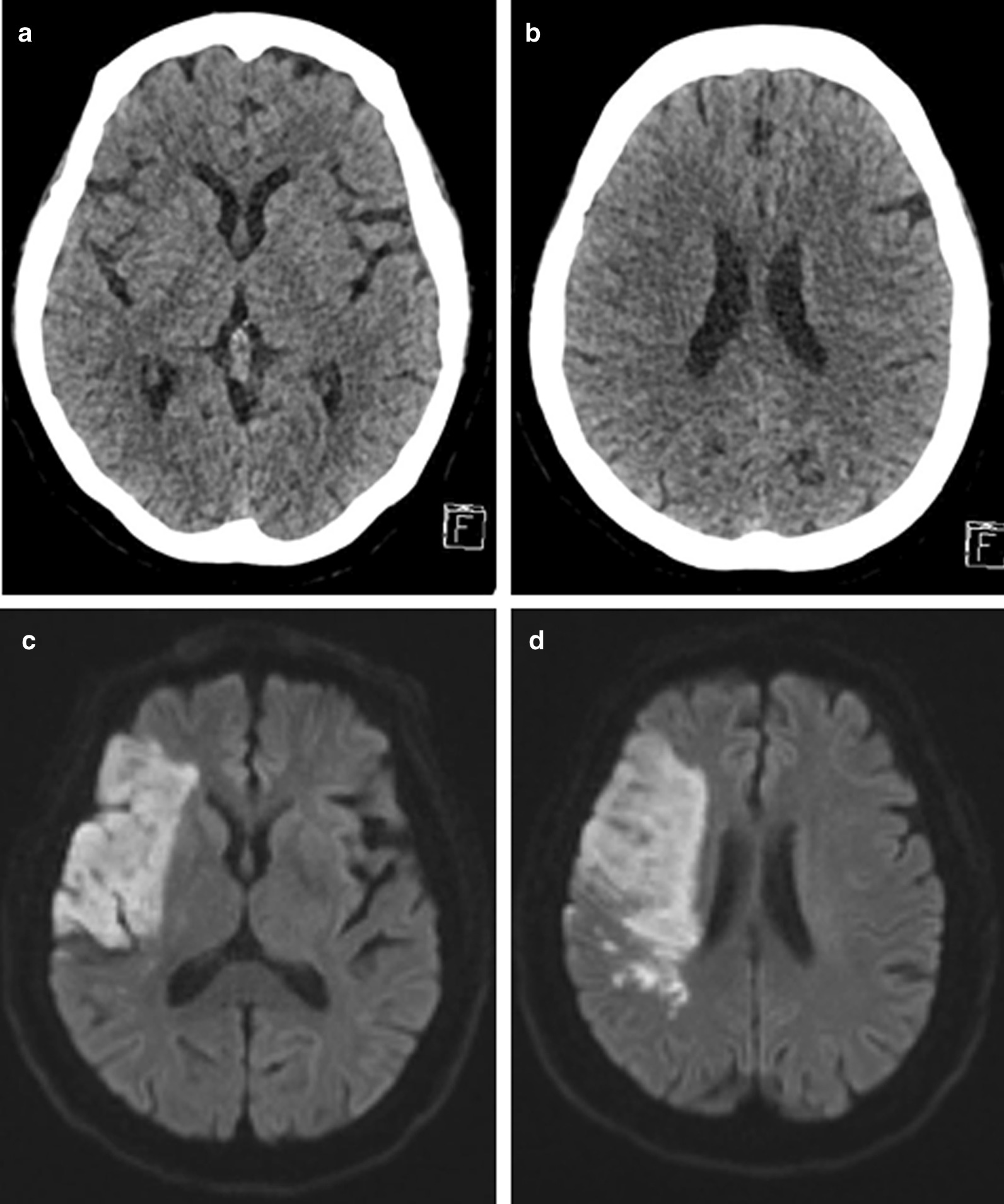
Patients with poor clinical prognosis: a patient was admitted to the hospital 277 min after the onset of stroke. At admission, the patient's ASPECTS was 9 points, and the HU difference was 16.01. The calculated on the DWI image at 7 days after thrombolysis was 231.95, and the 3-month mRS score was 4 points. **a**, **b** NCCT image on admission. **c**, **d** DWI image at 7 days after thrombolysis

The ASPECTS is a scoring system for evaluating early ischemic changes in the blood supply area of the middle cerebral artery by NCCT. It is essentially based on visually low attenuation. In addition, the ASPECTS is time-dependent, and a study shown that the reliability of ASPECTS is moderate at stroke onset time < 90 min, good at 90–180 min, and excellent at > 180 min. The HU value is based on the attenuation coefficient of water to calculate the HU value of each tissue [17], which is quantitative in nature, and the measurement of HU value is independent of the subjective interpretation of any observer. Compared with the ASPECTS, the reliability and consistency of the HU value are greatly improved. In this study, two patients had the same ASPECTS, but their HU values changed differently, and their clinical outcomes were different (see Figs. 4, 5). The ROC curve showed that the AUC of the HU ratio was 0.743, when the cutoff value was 10, it had a higher sensitivity of 80.56%. The AUC of the HU difference was 0.833, when the cutoff value was 13.97, it had a higher specificity of 93.75%. The results indicated that the HU ratio and HU difference had important predictive value for the prognosis of stroke patients treated by IVT. For stroke patients with the same ASPECTS, the HU value can be used for a second evaluation to improve the accuracy of diagnosis. In the future, more research is needed to perfect this algorithm to improve the predictive value of HU. Our study has some limitations: ① The influence from the occlusion position on the patient was not taken into account, for stroke patients with a distant occlusion position, the infarct size was too small, and the HU value did not change significantly. ② Previous study shown that the weight of each area of ASPECTS was different [18]. We did not weight each area when calculated the HU ratio or HU difference. ③ The sample size was small. ④ No subgroup analysis based on ASPECTS. ⑤ Ten ASPECTS regions were manually outlined and maybe there is an deviation. ⑥ The HU difference or ratio per patient without error used subsequent research and analysis may cause statistical error. In the future, we will adopt imageomics or deep learning methods to avoid it.

## Conclusion

In summary, the HU value changes are related to the clinical prognosis of stroke patients with MCAO treated by IVT, HU value may be a prognostic indicator for stroke patients with MCAO treated by IVT.

## Data Availability

The datasets used during the current study are available from the corresponding author on reasonable request.

## Abbreviations

ASPECTS: Alberta Stroke Program Early CT Score
AUC: Area under receiver operating characteristic curve
HMCAS: Hyperdense middle cerebral artery sign
HU: Hounsfield unit
IVT: Intravenous thrombolysis
LNO: Lenticular nucleus obscuration
MCAO: Middle cerebral artery occlusion
mRS: Modified Rankin Scale
NCCT: Non-contrast computed tomography
NIHSS: National Institute of Health stroke scale
OML: Orbito-meatal line
